# Article 2: Longitudinal study assessing the one-year effects of supervision performance assessment and recognition strategy (SPARS) to improve medicines management in Uganda health facilities

**DOI:** 10.1186/s40545-018-0142-1

**Published:** 2018-07-05

**Authors:** Birna Trap, Richard Musoke, Anthony Kirunda, Martin Olowo Oteba, Martha Embrey, Dennis Ross-Degnan

**Affiliations:** 1 USAID/Uganda Health Supply Chain Program, Management Sciences for Health, Plot 15, Princess Anne Drive, Bugolobi, P.O. Box 71419, Kampala Uganda; 2 Management Sciences for Health, Plot 15, Princess Anne Drive, Bugolobi, P.O. Box 71419, Kampala Uganda; 3 Ministry of Health Uganda, Pharmacy Department, Plot 6/Lourdel Rd, P.O. Box 7272, Kampala, Uganda; 4 Management Sciences for Health, 4301 N. Fairfax Drive, Suite 400, Arlington, VA 22203 USA; 5 Harvard Medical School and Harvard Pilgrim Health Care Institute, 401 Park Drive Suite 401, Boston, MA 02215 USA

**Keywords:** Supportive supervision, Medicines management interventions, Multipronged intervention, Performance assessment, Public sector, Uganda, Supply chain, Medicines use, Medicines indicators

## Abstract

**Background:**

In late 2010, Uganda introduced a supervision, performance assessment, and recognition strategy (SPARS) to improve staff capacity in medicines management in government and private not-for-profit health facilities. This paper assesses the impact of SPARS in health facilities during their first year of supervision.

**Methods:**

SPARS uses health workers trained as Medicines Management Supervisors (MMS) to supervise health facilities and address issues identified through indicatorbased performance assessment in five domains: stock management, storage management, ordering and reporting, prescribing quality, and dispensing quality. We used routine data generated during SPARS visits to 1222 health facilities to evaluate performance changes during the first year of supervision as well as the time until achieving an adequate score in this period. We also explored variables related to facilities, MMS, and intensity of implementation as predictors of performance improvement and time until achieving an adequate score.

**Results:**

Health facilities received an average of 3.4 MMS visits during the first year of supervision, with an average of 88 days between visits; each MMS implemented a median of 28 visits per year. Overall SPARS scores (maximum of 25) improved by 2.3 points (22.3%) per visit from a mean baseline score of 10.3. The adjusted improvement in overall SPARS score was significantly higher in primary health care facilities (2.36) versus higher-level health facilities and hospitals (2.15) (*p* = 0.001). The incremental improvement was highest at visit 2, with decreasing but continuing positive gains in subsequent visits. The adjusted mean incremental improvement per visit was highest in the prescribing quality domain, followed by dispensing quality, ordering and reporting, stock management, and storage management. Adjusted improvement in SPARS scores varied by region, year of implementation, and facility ownership. After one year of SPARS, 22% of facilities achieved an adequate score of 18.75 (75% of maximum score).

**Conclusions:**

SPARS was effective in building health facility capacity in medicines management, with a median overall improvement of almost 70% during the first year. The greatest improvements occurred in prescribing quality and at lower levels of care, although the highest level of performance was achieved in storage management. We recommend broad dissemination of the SPARS approach in all Ugandan health facilities as well as in other countries seeking a practical strategy to improve medicines management performance.

**Electronic supplementary material:**

The online version of this article (10.1186/s40545-018-0142-1) contains supplementary material, which is available to authorized users.

## Background

In Uganda, the Ministry of Health’s (MOH) Pharmacy Department implemented a new national strategy that reorganizes health services around patients’ needs and coordinates relationships between essential medicines and health supplies (EMHS) and other health system components to increase responsiveness and produce better outcomes [1]. As part of this focus on responsiveness and accountability, facilities need to be able to optimize available resources and meet growing expectations for better performance in medicines management (MM).

Effective MM in health care delivery involves many stakeholders and systems, and requires the optimization of processes covering five domains: stock management, storage management, ordering and reporting, prescribing quality, and dispensing quality [2]. Barriers to effective MM are many, complex, and interconnected, which calls for a holistic health system improvement approach [3]. Previously, Uganda had implemented predominantly educational interventions in health facilities, though with limited and unsustainable impact [4–6]. In late 2010, Uganda’s MOH began to pilot a Supervision, Performance Assessment and Recognition Strategy (SPARS) to improve MM in health facilities, an approach that uses supportive supervisory visits, indicator-based performance assessment, sharing performance findings with managers at all levels, and special recognition for good performance. This multi-pronged approach is based on evidence on best practices in achieving sustainable health system performance improvements [7–9]. The cornerstones of SPARS are the Medicines Management Supervisors (MMS), who in addition to supportive supervision, also provide managerial support to staff in the form of manuals and tools needed to standardize MM practices. The MMS use SPARS indicators measured during each visit to identify weak areas and focus attention using effective supportive supervision principles [2, 10–12]. The SPARS method is described in detail in the first article of this theme issue [2].

To assess the longitudinal impact of the SPARS program, which was rolled out nationally in 2012, we assessed performance results during the first year of supervision in government and private not-for-profit (PNFP) health facilities initiated in the program from the end of 2010 through 2013. Facilities represented all levels of care and came from 45 districts, representing about half of Uganda’s districts.

## Methods

### Study design

This was an indicator-based longitudinal prospective study assessing incremental changes in SPARS scores, both overall and by MM domain, from the initial MMS visit through to the last visit conducted during the first year of supervision in each facility.

### Setting and context

Uganda had a 2013 population of close to 38 million with an annual growth rate of 3.2% per year [13]. In that year, health care services were provided in the then 116 districts through 6404 health facilities, of which 63% (4035) were public (comprising 48% [3074] government-owned and 15% [961] PNFP) and 37% (2369) were private for-profit [13]. Service levels range from health center 1 (HC1), which represents volunteer health teams rather than actual facilities, to national referral hospitals. Each level of health facility is intended to supervise the level below. Table 1 lists the number of government and PNFP facilities and service levels in Uganda.

**Table 1:** Government and PNFP health facilities and services by level of care in Uganda in 2017

	HC1	HC2	HC3	HC4	General hospital	Regional referral hospital	National referral hospital
Total number	25,000	2354	1291	196	117	16	2
Population served	1000	5000	20,000	100,000	500,000	2 million	10 million
Service area	Village	Parish	Sub-county	Sub-district	District	Regional	National
Staffing	Village health workers	Nurses	Clinical officers, nurses	Doctors, clinical officers, nurses	Doctors, clinical officers, pharmacy technicians, nurses	Specialists, doctors, clinical officers, pharmacists nurses	Specialists, doctors, clinical officers, pharmacists nurses
Services	Preventive; health promotion; reproductive, maternal, newborn, child health	Preventive; promotion; outpatient curative; maternity; community outreach and emergencies	HC2 plus: inpatient health services; simple diagnostic/laboratory services	HC3 plus: emergency surgery; blood transfusion; laboratory services	HC4 plus: service training; consultation; research	General hospital plus: specialist’s services such as psychiatry, ear, nose, and throat, ophthalmology, dentistry, intensive care, radiology, pathology, and more complex surgery	Regional referral hospital plus: specialists’ services; training and research

When SPARS was introduced, the average availability of a basket of 22 vital items in public health facilities was 53% on the day of survey, and providers at only 1% of health facilities provided the correct treatment for simple cough and cold [4]. Moreover, less than 8% (31) of pharmacy posts in the public sector were filled [14], and the health services referral system was poorly implemented [15].

Government hospitals and HC4s order their medicines and supplies, while HC2s and HC3s receive pre-packed kits; PNFP facilities at all levels order their supplies. Government facilities provide EMHS free of charge, which resulted in US$2.40 per capita spending for EMHS in 2013/14; the supply is heavily dependent on donor funds, which covered 77% of EMHS costs in 2013/14 [14].

### SPARS intervention and its components

MMS are health sector employees such as clinical officers, nurses, EMHS storekeepers, or pharmacy staff who are trained to make SPARS supervisory visits along with their other duties. MMS are selected by district health officers. Each district has one MMS who supervises mainly higher-level facilities (HC4 and hospitals) and oversees two to five health sub-district (HSD) MMS, who supervise lower-level facilities (HC3 and HC2). MMS are given motorbikes for transportation; netbooks and modems to submit facility performance assessment data to a central information platform; MM tools such as stock cards, dispensing logs, and manuals describing standard operating procedures; and job aids and recognition materials for health facilities. The MMS are reimbursed US$12 for each assessment report they submit.

The MMS carry out the following activities to implement SPARS:

- Inform facilities in advance about upcoming SPARS visits
- Orient facility staff on the visit’s purpose and conduct the indicator-based performance assessment
- Discuss assessment findings with health facility staff to highlight indicators that have improved, to see if targets have been met, and to identify problems
- Follow up with mentoring and training sessions that focus on skills or procedures that need improvement
- Agree with the facility staff on tasks to complete for the next visit
- Debrief health facility staff and facility in-charge about the visit
- Fill out the SPARS supervisory book with SPARS indicator scores for the current visit and targets for the next visit
- Fill in the SPARS data collection electronic tool [2] and submit to the central database

District and HSD MMS are each expected to complete three and five supervisory visits per month, respectively. Optimally, after the initial assessment, MMS should visit facilities every 60 days until they achieve an acceptable SPARS score (see below); after that, the maintenance phase of the program calls for three (3) visits per facility per year.

### Sampling and data sources

For this study, we randomly selected 45 of the 80 districts included in the SPARS implementation from the end of 2010 to mid-2013, representing 15, 13, 9, and 8 districts from the Western, Eastern, Northern, and Central regions, respectively. Data for this study were results from SPARS performance assessments extracted from the centralized data platform for all visits that occurred for a period of 1 year after the initial visit to each facility. The data for the performance assessments came from stock management records, receipt and issue vouchers, dispensing logs, and laboratory logs. MMS also observed staff practices and the facility environment, and conducted exit interviews to assess patient knowledge and medicine labeling.

### Outcome variables

#### SPARS overall, domain scores and achievement of adequate scores

The 25 SPARS indicators are classified into five MM domains: dispensing quality (seven indicators); prescribing quality (five indicators); stock management (four indicators); storage management (five indicators); and ordering and reporting (four indicators). Each of the five domains is assigned a maximum score of 5, resulting in a maximum overall SPARS score of 25. Each indicator is weighted proportionally to its contribution to the domain score, with missing indicators removed from the weighted domain score calculation. We defined an “adequate” SPARS score to be 18.75, equal to 75% of the maximum score.

#### Assessment of change in SPARS and domain scores

The primary outcome measure in this study was the change in total SPARS score between each pair of successive visits that took place during the first year of follow-up in each facility; changes in individual domain scores were secondary outcomes. We also assessed the median number of visits per facility and the median number of days between visits in the follow-up year. Finally, we determined whether a facility achieved an adequate SPARS score of 18.75 at any time during the follow-up year, as well as the time it took for the facility to reach this score.

#### Predictor variables

We identified two categories of predictor variables. Facility characteristics, which were assessed for all study facilities from either administrative data or from SPARS visit records, included: level of care (HC2, HC3, HC4, or hospital); ownership (government or PNFP); region (Eastern, Western, Northern, Central); calendar year of the initial SPARS visit; number of SPARS visits in the follow-up year; number of health facility staff supervised in the initial visit (one or more than one); number of MMS supervising at the initial visit (one or more than one); and whether the MMS who conducted the initial visit was assigned to the facility (yes or no). Because of differences in staffing, supply ordering, and services delivered, we stratified facilities by level of care for all analyses, with HC4 and hospitals grouped together at the highest level of care.

For each visit, we also assessed key characteristics of the MMS who conducted the visit including: gender; level (district or HSD); professional training (doctor/clinical officer, pharmacist/dispenser, nurse/midwife, supply officer); and number of facilities assigned to the MMS. For 74.5% of visits, we linked results of a survey completed in 2013 that included data on age, highest level of education, number of years of work experience, frequency of meeting with the District Health Officer (DHO), whether the MMS received feedback from the DHO about reports, whether the MMS felt that there was sufficient time to provide adequate supportive supervision during a visit, and whether the MMS felt that health workers responded well to the supervision.

#### Imputation

Based on data from completed SPARS visits, we employed multiple imputation methods to impute values of missing survey predictors for use in regression models [16, 17]; we also imputed values for missing SPARS domain scores.

### Statistical analysis

We used chi-square tests to compare characteristics of facilities and MMS by level of health facility. Mean, median, and interquartile ranges (IQR) of overall SPARS and domain scores were calculated by follow-up visit number and compared across level of care. We examined changes in baseline scores during the initial SPARS visits in the period from 2011 to 2013 in order to examine possible temporal changes in scores unrelated to the intervention. We used generalized linear models with clustering on facility and MMS to assess the association between each individual predictor variable and the outcomes of interest. Predictors that were statistically significant in bivariate analyses were considered for multivariate analyses using the same models. Based on the estimates from the final multivariate models, we calculated adjusted values of the change scores along with their means and 95% confidence intervals. We displayed time until reaching an adequate SPARS score by level of care with Kaplan–Meier survival curves and used Cox-proportional hazard models to assess the time until attainment of an adequate score and the predictors of this outcome. Multiple imputation of missing data and all statistical analyses were conducted using STATA version 13.1.

## Results

### Characteristics of health facilities and visits

MMS visited 1499 facilities between 2010 and 2013 in the 45 sample districts; due to lost or incomplete reports, 1384 facilities (92%) had an analyzable record available for their initial assessment, and 1222 (82%) had at least one follow-up visit in the 12 months after their initial visit and were included in the analysis. Overall, 85% were government and 15% were PNFP facilities, and the analyses included 681 HC2s (56%), 416 HC3s (34%) and 125 HC4s and general hospitals (10%) (Table 2).

**Table 2:** Facility and visit characteristics

Study facilities	Total		HC2		HC3		HC4/hospital		*χ* ^2^
	No.	%	No.	%	No.	%	No.	%	p-value
	1222	100	681	100	416	100	125	100	
Region									
Central	250	21	133	20	92	22	25	20	0.343
Western	421	35	224	33	145	35	52	42	
Eastern	379	31	226	33	118	28	35	28	
Northern	172	14	98	14	61	15	13	10	
Ownership									
Government	1039	85	596	88	349	84	94	75	0.002
PNFP	183	15	85	13	67	16	31	25	
Year of initial visit									
2011	753	62	368	54	289	70	96	77	<0.001
2012	406	33	263	39	117	28	26	21	
2013	63	5	50	7	10	2	3	2	
Health workers supervised at initial visit									
One	280	23	223	33	45	11	12	10	<0.001
More than one	942	77	458	67	371	89	113	90	
MMS supervising during initial visit									
One	957	78	603	89	292	70	62	50	<0.001
More than one	265	22	78	12	124	30	63	50	
Designated MMS supervised initial visit^a^									
No	394	32	208	31	150	36	36	29	0.118
Yes	828	68	473	69	266	64	89	71	

^a^Designated MMS is the MMS assigned to a facility who was responsible for a majority of visits

Facilities were comparable across levels of care by region. Lower-level facilities had higher percentages of government ownership (*p* = 0.002) and fewer had started SPARS supervision in 2011 (*p* < 0.001). At the initial visit, a greater percentage of HC2s were supervised by only one MMS (*p* < 0.001) and higher-level facilities had a greater percentage of initial visits in which two or more health workers were supervised (*p* < 0.001). The designated MMS for a facility conducted the initial supervision in about two-thirds of facilities.

### Characteristics of medicines management supervisors

Of the 148 MMS included in the study, 84% (124) were male, 64% (95) were HSD level, 55% (81) supervised 10 facilities or fewer, and 59% (87) were trained as clinical officers (Table 3). A total of 111 of the 148 MMS (75%) included in the study completed the 2013 MMS characteristic survey. Of these, 42% (46) were age 36 to 45, 83% (92) had secondary or diploma level education, and 40% (45) had fewer than 10 years of experience. The majority of MMS completing the survey reported having a monthly or weekly meeting with the DHO, and 85% (92) received feedback from the DHO on their submitted reports. About two-thirds of MMS felt they had sufficient time for conducting supervision during visits, and two-thirds thought that health workers responded well to the supervision (Table 3).

**Table 3:** Medicines management supervisor and district health officer characteristics

Characteristics	No.	%
MMS study total	148	100
Gender		
Male	124	84
Female	24	16
Level		
District MMS	53	36
Sub district MMS	95	64
Regions		
Central	31	21.0
Western	56	37.8
Eastern	41	27.7
Northern	20	13.5
Facilities supervised		
1-10	81	54.7
11-15	47	31.7
16+	20	13.6
Professional training		
Clinical officer	87	59
Pharmacist/dispenser	15	10
Nurse	36	24
Supplies officer	10	7
MMS completing 2013 survey	111	75
Age group		
26-35	37	34
36-45	46	42
46+	26	24
Highest level of education		
Secondary/diploma/other	92	83
Bachelors/Master’s degree	19	17
Number of years of work experience		
0-9	45	40
10+	66	60
Frequency of meetings with DHO		
Monthly/weekly	60	54
Quarterly/semi-annually	24	22
Irregularly/other	27	24
Received feedback from DHO about MMS report		
No	16	15
Yes	92	85
Sufficient time during visits to provide adequate supportive supervision		
No	38	35
Yes	71	65
Health workers respond well to supervision		
Some of them	40	37
Most/all of them	68	63

### Intensity of intervention implementation

In the 1222 health facilities, MMS carried out 4172 supervisory visits in the first year of supervision with an average of 3.4 visits per facility. The median number of visits per facility was 3 (IQR 2–4), and the median number of days between visits was 88 (IQR 61–132). The median number of visits per year per designated MMS was 28 (IQR 17–39) (Table 4).

**Table 4:** Number of MMS visits within the first year of supervision, overall and by level of care

No. of visits	All facilities		HC2		HC3		HC4/hospitals	
	No.	%	No.	%	No.	%	No.	%
2	328	27	184	27	115	28	29	23
3	334	27	176	26	115	28	43	34
4	323	26	180	26	108	26	35	28
5	201	16	122	18	62	15	17	14
6	35	3	19	3	15	4	1	1
7	1	0	0	0	1	0	0	0
Total	1222	100	681	100	416	100	125	100

### Changes in SPARS scores over time, overall, by level of care, and by domain

**Fig. 1:**
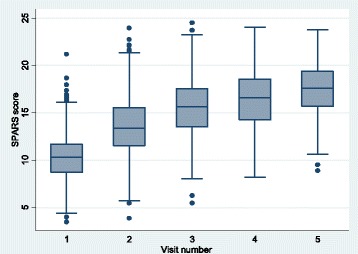
Overall SPARS scores by visit during the first year, indicating median (middle line), percentiles (25th and 75th shaded area, 5th and 95th outer lines), and extreme values

**Fig. 2:**
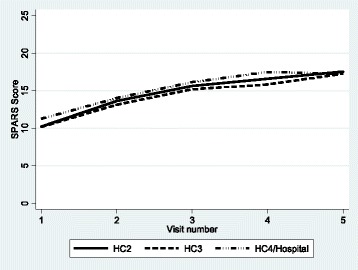
Mean overall SPARS scores by visit during the first year, by level of care

**Fig. 3:**
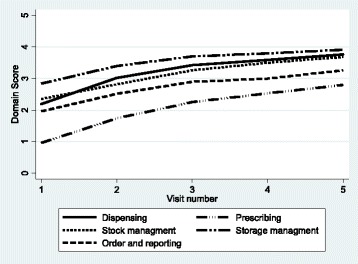
Overall domain score by visits

The median overall SPARS score increased by 68.9% from 10.3 (IQR 8.7–11.7) at the initial visit to 17.4 (IQR 15.6–19.4) at visit 5 (Fig. 1). The median improvements in SPARS score declined with each succeeding visit during the first year. The mean overall SPARS scores were slightly higher in HC4s and hospitals and slightly lower in HC3s at all visits, but improvements in SPARS scores by visit were very similar across all levels of care (Fig. 2). The initial visit domain scores and the improvement over time differed by MM domain. Storage management had the highest mean score at the initial visit (baseline) of 2.8 (95% CI 2.75–2.85), while the prescribing quality domain had the lowest mean of 1.0 (0.93–1.00). By visit 5, the mean domain scores were all above 3.0 except for prescribing quality at 2.8 (2.65–2.94); however, prescribing quality experienced the greatest absolute improvement during the course of follow-up (Fig. 3).

The average adjusted baseline SPARS score in the study facilities prior to any intervention in 2010 to 2011 was 10.25, which improved to 10.57 in 2012 and to 11.29 in 2013. This represented 0.32 and 1.04 point improvements in baseline scores in 2012 and 2013, respectively, unrelated to the SPARS intervention.

### Improvement in SPARS scores by visit

Table 5 presents the average changes in SPARS scores by level of care, overall and by domain, adjusted for the predictors included in the multivariate models. Averaged over all visits in the first year of supervision, the adjusted improvement in SPARS score per visit was slightly but significantly higher in HC2s (2.2) compared to hospitals or HC4s (2.0). The adjusted mean improvement in SPARS scores was highest at visit 2 (i.e., following the initial supervision) at all facility levels, but the improvement was significantly higher in HC2s and HC3s (3.2 and 2.8, respectively) than in higher-level facilities (2.5). Across all the three levels of care, the adjusted mean improvements were lower at visit 3 (after two rounds of supervision) and lower still at visit 4. The numbers of facilities with a fifth supervisory visit in the first year were low at all levels of care, but among those with a fifth visit, changes in adjusted overall SPARS scores remained positive.

**Table 5:** Adjusted ^a^ mean change in overall SPARS and domain scores by level of care and visit number during the first year of supervision

HC2			HC3		HC4/hospital		All facilities	
Observations		Adj. Diff. (95%CI)	Observations	Adj. Diff. (95%CI)	Observations	Adj. Diff. (95%CI)	Observations	Adj. Diff. (95%CI)
Total SPARS score								
All	1639	2.2 (2.14 - 2.27)	980	2.1 (2.04 - 2.19)	292	2.0 (1.87 - 2.11)	2911	2.2 (2.11 - 2.20)
2	679	3.2 (3.07 - 3.26)	416	2.8 (2.68 - 2.87)	125	2.5 (2.34 - 2.66)	1220	3.0 (2.90 - 3.02)
3	496	2.1 (1.97 - 2.19)	301	2.1 (1.87 - 2.25)	96	2.1 (1.85 - 2.42)	893	2.1 (2.01 - 2.15)
4	323	0.8 (0.72 - 0.95)	186	1.0 (0.75 - 1.16)	53	1.1 (0.91 - 1.28)	562	0.9 (0.82 - 0.97)
5	141	1.2 (1.03 - 1.33)	77	1.6 (1.31 - 1.93)	18	0.3 (-0.01 - 0.68)	236	1.3 (1.15 - 1.36)
Stock management								
All	1635	0.3 (0.31 - 0.36)	980	0.4 (0.34 - 0.40)	292	0.4 (0.29 - 0.42)	2907	0.4 (0.32 - 0.38)
2	679	0.4 (0.37 - 0.45)	416	0.5 (0.40 - 0.53)	125	0.4 (0.24 - 0.50)	1220	0.4 (0.38 - 0.47)
3	494	0.4 (0.36 - 0.43)	301	0.4 (0.31 - 0.42)	96	0.5 (0.40 - 0.56)	891	0.4 (0.37 - 0.42)
4	321	0.1 (0.10 - 0.18)	186	0.3 (0.20 - 0.32)	53	0.2 (0.04 - 0.35)	560	0.2 (0.16 - 0.21)
5	141	0.2 (0.15 - 0.34)	77	0.2 (0.04 - 0.35)	18	0.1 (-0.22 - 0.42)	236	0.2 (0.15 - 0.28)
Storage management								
All	1639	0.4 (0.34 - 0.38)	980	0.4 (0.33 - 0.39)	292	0.3 (0.23 - 0.30)	2911	0.3 (0.34 - 0.36)
2	679	0.6 (0.51 - 0.59)	416	0.5 (0.45 - 0.61)	125	0.4 (0.29 - 0.53)	1220	0.5 (0.50 - 0.57)
3	496	0.4 (0.29 - 0.43)	301	0.3 (0.21 - 0.34)	96	0.2 (0.05 - 0.29)	893	0.3 (0.28 - 0.34)
4	323	0.1 (0.03 - 0.11)	186	0.1 (0.06 - 0.23)	53	0.2 (0.12 - 0.35)	562	0.1 (0.08 - 0.14)
5	141	0.1 (-0.03 - 0.23)	77	0.2 (0.13 - 0.33)	18	-0.1 (-0.27 - 0.06)	236	0.1 (0.05 - 0.20)
Order reporting								
All	1639	0.4 (0.34 - 0.39)	980	0.3 (0.29 - 0.35)	292	0.5 (0.46 - 0.58)	2911	0.4 (0.35 - 0.38)
2	679	0.5 (0.42 - 0.52)	416	0.3 (0.29 - 0.41)	125	0.7 (0.62 - 0.83)	1220	0.5 (0.42 - 0.48)
3	496	0.4 (0.30 - 0.40)	301	0.4 (0.32 - 0.45)	96	0.5 (0.32 - 0.60)	893	0.4 (0.33 - 0.42)
4	323	0.2 (0.12 - 0.25)	186	0.1 (0.01 - 0.22)	53	0.2 (0.05 - 0.38)	562	0.2 (0.11 - 0.22)
5	141	0.3 (0.24 - 0.40)	77	0.5 (0.39 - 0.58)	18	0.3 (-0.02 - 0.58)	236	0.4 (0.30 - 0.44)
Prescribing quality								
All	1639	0.6 (0.59 - 0.62)	980	0.6 (0.52 - 0.60)	292	0.4 (0.38 - 0.45)	2911	0.6 (0.56 - 0.58)
2	679	0.8 (0.76 - 0.80)	416	0.7 (0.65 - 0.81)	125	0.5 (0.41 - 0.54)	1220	0.7 (0.70 - 0.75)
3	496	0.6 (0.60 - 0.66)	301	0.5 (0.49 - 0.57)	96	0.5 (0.44 - 0.58)	893	0.6 (0.56 - 0.60)
4	323	0.3 (0.26 - 0.33)	186	0.3 (0.24 - 0.37)	53	0.2 (0.12 - 0.35)	562	0.3 (0.27 - 0.32)
5	141	0.4 (0.33 - 0.43)	77	0.4 (0.35 - 0.51)	18	0.1 (-0.19 - 0.30)	236	0.4 (0.32 - 0.42)
Dispensing quality								
All	1639	0.5 (0.52 - 0.56)	980	0.5 (0.48 - 0.52)	292	0.4 (0.40 - 0.46)	2911	0.5 (0.51 - 0.53)
2	679	1.0 (0.95 - 0.98)	416	0.7 (0.68 - 0.73)	125	0.5 (0.48 - 0.56)	1220	0.8 (0.82 - 0.84)
3	496	0.3 (0.33 - 0.36)	301	0.5 (0.48 - 0.54)	96	0.5 (0.47 - 0.55)	893	0.4 (0.40 - 0.43)
4	323	0.1 (0.12 - 0.16)	186	0.1 (0.09 - 0.16)	53	0.2 (0.16 - 0.28)	562	0.1 (0.13 - 0.16)
5	141	0.1 (0.11 - 0.17)	77	0.3 (0.23 - 0.33)	18	0.0 (-0.08 - 0.1)	236	0.2 (0.15 - 0.20)

^a^Models adjusted for baseline SPARS scores and the significant predictors in the individual multivariate analyses, which are listed for each model in Table 6 and Appendix 1

Across the five indicator domains, improvements in SPARS scores tended to follow a similar pattern with the largest improvements observed at visits 2 and 3, and smaller gains observed in later visits. Across all visits, average improvements in prescribing quality scores were notably lower in HC4s and hospitals (0.4) than in HC2s and HC3s (0.6 each). The average adjusted improvements in the first year in the prescribing domain were the highest of any domain. For HC4s and hospitals, the largest adjusted improvements in any domain were observed for ordering and reporting (0.5), with a particularly large gain observed after the first visit (0.7) (Table 5).

### Predictors of improvement in SPARS and domain scores

In addition to the baseline score, the factors significantly associated with average visit-to-visit improvement in overall SPARS scores at all facilities in multivariate models included region, ownership, the number of MMS supervising a facility at the previous visit, MMS profession, and whether the MMS received feedback from the DHO (Table 6). Specifically, adjusting for the level of the baseline SPARS score, significantly greater improvements were observed in the Northern (0.8 greater improvement, 95% CI [0.55, 1.01]), Western (0.5, [0.32, 0.72]), and Eastern (0.3, [0.13, 0.51]) regions compared to the Central region, with differences primarily in lower level health facilities. Greater changes were observed when more than one MMS supervised a facility (0.3, [0.02, 0.63]), driven primarily by performance in HC4 and hospitals (0.9, [0.21, 1.58]). MMS who were pharmacists or dispensers tended to be associated with higher overall improvements in SPARS scores compared to other professions, and facilities supervised by storekeepers experienced significantly lower improvements (− 0.7, [− 1.04, − 0.35]) than those supervised by pharmacists. Significantly greater improvement in overall SPARS scores occurred in facilities supervised by MMS who were supported by an engaged DHO who provided feedback on the SPARS reports to the MMS (0.6, [0.30, 0.95]).

**Table 6:** Results of multivariable models showing factors significantly associated with average changes in the overall SPARS scores by level of care and in all facilities

	HC2		HC3		HC4/Hospital		All facilities	
	Observations	Adj. Diff. (95%CI)	Observations	Adj. Diff. (95%CI)	Observations	Adj. Diff. (95%CI)	Observations	Adj. Diff. (95%CI)
Region								
Central	290	—	208	-			554	-
Western	493	**0.3 (0.00, 0.55)**	285	**0.7 (0.42, 1.04)**			883	**0.5 (0.32, 0.72)**
Eastern	625	0.2 (-0.09, 0.43)	365	**0.5 (0.16, 0.76)**			1087	**0.3 (0.13, 0.51)**
Northern	231	**0.7 (0.35, 0.96)**	122	**0.9 (0.46, 1.29)**			383	**0.8 (0.55, 1.01)**
Ownership								
Government	1434	—					2472	—
PNFP	205	**-0.4 (-0.69, -0.13)**					435	-0.2 (-0.39, 0.01)
Number of MMS supervising a facility								
One MMS					196	—	2541	—
More than one MMS					96	**0.9 (0.21 , 1.58)**	366	**0.3 (0.02, 0.63)**
Number of facilities MMS is designated								
1-10	578	—						
11-15	724	**0.2 (0.06, 0.44)**						
16+	337	**0.3 (0.05, 0.61)**						
Profession of responsible MMS								
Pharmacist/dispensers	135	—					302	—
Clinician	928	**-0.4 (-0.75, -0.01)**					1618	-0.2 (-0.47, 0.01)
Nurse/midwife	478	**-0.4 (-0.83, -0.06)**					789	-0.2 (-0.46, 0.05)
Storekeeper	98	**-0.8 (-1.33, -0.29)**					198	**-0.7 (-1.04, -0.35)**
Received feedback from DHO about MMS report								
No			162	—			414	—
Yes			818	**0.7 (0.32, 1.17)**			2493	**0.6 (0.30, 0.95)**
**Baseline score**	1639	**-0.2 (-0.29, -0.21)**	980	**-0.3 (-0.33, -0.21)**	292	**-0.2 (-0.3, -0.13)**	2907	**-0.3 (-0.28, -0.22)**

Figures in bold indicate adjusted differences with 95%CIs that did not include zero based on the multivariable models

Additional file 1 shows the factors that are significantly associated with improvements in the individual SPARS domain scores by level of care. Notably, improvements in the prescribing indicators were significantly higher when the MMS was a clinical officer or nurse in HC3, and at all facility levels, MMS who were trained as storekeepers had significantly lower impact on prescribing. Improvements in ordering and reporting and in stock management were higher when the MMS had a pharmaceutical background, and stock management improvements were significantly higher when the MMS received regular supervision and more than one health worker was supervised.

### Time and number of visits to reach adequate score

**Fig. 4:**
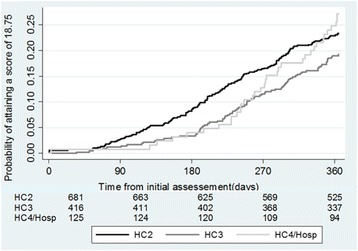
Number of days to attain SPARS score of 18.75 by level of care

A total of 273 (22%) out of 1222 facilities attained an adequate score of 18.75 in the first year of supervision (Fig. 4). A greater proportion of HC2s achieved an adequate score earlier in the year, but the proportion of HC4s and hospitals performing at this level surpassed them by the end of the follow-up year; HC3s had the lowest proportion of adequately performing facilities. Of all facilities achieving an adequate score, the median number of days to reach that level of performance was 234 (IQR 173–294).

Adjusting for whether a facility was above or below the baseline mean level of SPARS performance, factors that significantly influenced whether the facility reached an adequate score in the first year of SPARS supervision included: greater number of visits, region, MMS profession, and receiving feedback from the DHO (Additional file 2). Specifically, health facilities were significantly more likely to achieve an adequate score in the fourth visit or later (hazard ratio = 3.0 [2.29,3.93]) and facilities in the Northern (3.7 [2.28,6.16]), Western (2.5 [1.49,4.06]), and Eastern (2.2 [1.36,3.58]) regions reached this standard more rapidly than those in the Central region; facilities supervised by clinical officers reached an acceptable score significantly faster than those supervised by pharmacists (1.7 [1.20,2.51]), especially in HC2s; and facilities supervised by MMS that received feedback from the DHO about their reports reached their goals significantly faster (2.3 [1.30,4.00]) than those supervised by MMS who did not receive DHO feedback.

## Discussion

### Improvement in SPARS scores over time

With an average of 22.3% increase in overall SPARS scores per visit during the first year of supervision, our study documents that SPARS is an effective multi-pronged intervention to improve MM in all levels of health care in both government and PNFP sectors. Almost one in four facilities reached an adequate score within the first year. The SPARS intervention was associated with the greatest improvements after the first supervisory visit, although the gains in SPARS scores continued to be positive but tapering in subsequent visits.

### Comparison to other studies

Other studies of supervision interventions in low resource settings suggest a small positive effect of supervision [7, 8, 18], but most have not used a comprehensive intervention approach or estimated the relative improvement in performance between sequential visits. The observed improvements associated with the SPARS strategy of 22.3% per visit and 68.9% following four visits were very high, suggesting that multifaceted strategies may be more successful than supervision alone (7). A review of 30 interventions targeting prescribing practices demonstrated a median improvement relative to control of 18% (8). In comparison, we saw a 180% improvement in the prescribing quality domain during the first year of SPARS supervision. A supervisory intervention in Zimbabwe demonstrated a statistically significant improvement (7%) in supply chain management compared to control [19], while SPARS produced a 14% improvement (2.8 to 3.2) in the mean SPARS stock management score after the initial visit. The SPARS approach is more comparable to an intervention in the Philippines that combined supervision of midwives with follow-up visits using an indicator-based checklist for performance assessment at each visit [20]. The study found 24% improvement in scores following an average of 3.1 visits or a 7.7% score increase per visit compared to the SPARS score improvement of 22.3% per visit. The large magnitude of relative improvement in SPARS may be partly due to the low level of initial performance, but the continuing improvement in scores following subsequent visits suggests that SPARS may be an effective approach even after performance reaches a higher level.

### Level of care

MM performance improved at all levels of care, independent of service complexity and staffing, but with considerable individual facility variation. In addition, similar to other studies, we found that level of care influenced intervention effect [7]. The highest impact occurred at the lowest level of care, HC2, followed by HC3, HC4, and hospitals. HC2 facilities have only one staff person, so that supervision in that level of care is consistently provided one-on-one; moreover, the services provided are simpler and fewer compared to higher levels of care.

### Domains

The prescribing quality domain had the lowest initial mean scores, while storage management had the highest by almost three-fold. We observed improvements in all five domains, with the prescribing quality domain experiencing the largest incremental gain per visit followed by dispensing quality, ordering and reporting, stock management, and storage management. The improvement in all domains tended toward a score ceiling of 4 out of maximum 5 by the end of 1 year of visits.

Performance in the ordering and reporting domain proved to be the hardest to improve. Lower levels of care (HC2 and HC3) still received essential medicines kits; therefore, they did not submit orders and had no way to practice and maintain their related skills in this domain. Meanwhile, facilities that did place orders (HC4 and hospitals), were slow to adhere to a new order and delivery schedule introduced in 2010. However, following the initial SPARS visits to orient staff on the new practices, HC4s and hospitals experienced the largest initial improvement in ordering and reporting scores, demonstrating the usefulness of SPARS in accelerating the take up of the new order and delivery schedule. In addition, all facilities in the public and PNFP sectors are supposed to report monthly into Uganda’s health management information system. However, no incentives exist for timely and accurate reporting, and no feedback is provided to facilities on their reporting performance. Therefore, we recommend incorporating SPARS indicators in this domain that assess reporting quality and accuracy, and then providing regular feedback on that performance to health facilities.

The prescribing domain had the lowest initial SPARS domain scores, but also the highest adjusted improvement of all the domains within 1 year. However, the average improvement per visit was notably lower in HC4s and hospitals. At these higher levels of care, the number of prescribers and prescribing complexity makes it more difficult to increase capacity in all prescribers.

We believe that the initial rapid improvement in dispensing quality is linked to the SPARS recognition component which assured that facilities received dispensing tools such as counting trays, dispensing envelopes, and also adequate shelving that facilitated appropriate storage and dispensing practices.

### Regions

SPARS had the greatest impact on performance in facilities in the Northern region, especially in HC2s and HC3s. We think that previous civil unrest in the Northern region deprived the population of most health service improvement interventions; now, facilities in the area are eager to catch up and make full use of the opportunities offered. The reasons for variation in other regions are unclear.

### Facility ownership

We found government facilities to be more responsive to the SPARS intervention, with PNFP facilities having a significantly lower average increase in SPARS score per visit—0.4 points lower than public facilities. One explanation could be that the MMS initially chose their target facilities, and although PNFP facilities fall under the DHOs’ responsibility, MMS might have prioritized government facilities. Since then, the MOH has established and trained MMS from the four medical bureaus that oversee the PNFP facilities. Having dedicated PNFP MMS who can ensure sufficient supervision will especially benefit the HC2 PNFP facilities, which are typically weaker performers located in very remote areas.

### Supervision by more than one supervisor

SPARS has a practical training component where district MMS lead HSD-MMS through five supervisory visits until they are prepared to carry out their own visits. Because the district MMS oversee higher-level facilities, those facilities often received supervision from more than one MMS, unlike the HC2 and HC3 facilities that HSD-MMS oversee alone. In addition, MOH staff members accompany district MMS on HC4 and hospital visits as part of their hierarchical oversight structure. Having more than one MMS at supervisory visits benefited the higher-level facilities, particularly, because their pharmaceutical management functions are more complex—more services, more staff members, and more medicines. Not only can the MMS support each other, but they can split tasks and interact with more staff members. Revised SPARS procedures should consider having two MMS visit the higher-level facilities during the first two visits.

### Volume of facilities and supervisory visits

SPARS was designed to have MMS making five supervisory visits per month for 10 months a year, with each facility receiving about five MMS visits in the first year. After 1 year of regular supervision, we expected facilities to reach an adequate performance score; after this, the frequency of visits could be reduced to a maintenance level, with four to 6 months between supervisory visits. In practice, we found that MMS made 28 visits per year with 88 days between supervisory visits, and each facility received an average of only 3.4 visits per year. Though the greatest performance increases occurred within the first three visits, only 22% of the facilities reached an adequate score within the first year. The impact was in line with our expectations, but because of the lower level of implementation intensity, it will take longer to reach national SPARS coverage and for the majority of facilities to achieve adequate scores. Other studies have confirmed our findings that effects increase with multiple supervision visits [20] and that the interval between visits had no observable impact [18]. It is important to recognize that all MMS have these responsibilities added to their normal duties; therefore, realistically, MMS were only able to dedicate three to 4 days per month to SPARS supervision. Our findings suggested that visiting one facility per day is an appropriate target for MMS. Two-thirds of them felt that they had sufficient time to assess performance and implement supportive supervision.

Surprisingly, we found that MMS with responsibility for a greater number of facilities had a higher impact in improving MM. HSD-MMS generally had more than 10 facilities to supervise, but because they were mostly HC2 facilities, it may have been easier to improve simpler MM practices.

### MMS profession

The selection of MMS for the SPARS program is critical. The most important criteria are motivation, interest in the program, and being effective and supportive supervisors [21]. The supervisor’s profession also influenced impact; MMS with a clinical background were more successful in changing the staff’s prescribing behavior compared to pharmaceutical or storekeeping backgrounds; presumably they were viewed more as professional colleagues with an understanding of the complexity of diagnosing and prescribing according to standard treatment guidelines. On the other hand, MMS who were trained in pharmacy had more effect on performance in the stock management and ordering and reporting domains, where expertise in EMHS logistics gave them an advantage in explaining related standard operation practices. Storekeepers working as MMS who had a more limited logistics background were not as successful in improving performance in these domains. We concluded that MMS who were experienced had technical expertise in certain areas were better able to influence performance in those areas, which has been confirmed by other studies [10].

### DHO engagement

As expected based on other evidence [7], having a dedicated and engaged DHO that is interested in SPARS and MMS performance made a substantial difference in the improvements observed; therefore, we recommend finding ways to meaningfully engage the DHOs early and routinely in SPARS implementation in their districts.

### Study limitations

The 45 randomly selected study districts were included because they were targeted by the US Agency for International Development health system strengthening program in Uganda. However, they represented more than half of the 89 districts in the country at the time of the study and were selected based on diversity, regional representation, poverty, and need. We believe they provided a good cross-sectional representation of Uganda’s districts. As noted previously, the MMS chose facilities to target within the selected districts, which could have biased the study (e.g., the MMS could have given priority to government, better-performing, or closer facilities). However, we included over 80% of the facilities in the selected districts in the study, which limited the extent of this possible bias. The study facilities represented one-third of the government and PNFP facilities in Uganda, with government facilities slightly overrepresented (85% of the sample) compared to their actual proportion (76%) [13]. Despite the imbalance, we were still able to detect significant differences related to facility ownership.

Over the study period, new MMS joined the study, some left, and their overall level of experience increased—effects that might have influenced the degree and timing of impact; however, because this was a real-world study, we did not try to control for MMS longevity or experience. We saw wide variation between facilities in impact that may be due to unmeasured factors, such as MMS supportive supervisory skills [22] or facility staffing or resources. Another limitation related to the analysis of predictors of improvement was that we only had a 75% response rate for predictor data from MMS in the MMS survey despite several follow-up telephone calls. However, we were able to use multiple imputation methods to impute results for these missing surveys; results using only cases with complete data were essentially equivalent to those obtained using imputed data.

During the 12-month follow-up period, an almost equal number of facilities had two, three, and four supervisory visits, and only about half of the facilities had five or more visits as intended. This may have been linked to limitations on the number of visits that MMS could actually implement in a month. However, some facilities might have had more active MMS or have been located closer to the MMS place of work, which may have resulted in differential improvement.

Baseline SPARS scores improved slightly but significantly by 0.32 and 1.04 points in 2012 and 2013 compared to 2011, independent of the SPARS interventions. SPARS was implemented in facilities in a phased manner in all intervention districts, and facilities implemented later in the study period would have known about SPARS prior to their first visit. Thus, we cannot rule out the possibility that some contamination from earlier SPARS facilities may have led to a slight improvement in MM over time in all facilities in the district. Alternatively, other external factors in the health system may have led to improvements in the performance areas measured by SPARS. Ideally, we would have had a control group of facilities outside of the SPARS districts, but such a design was not feasible in the context of SPARS implementation. However, the types of consistent improvements in performance that we observed are most likely due in large part to the intervention rather than to other unobserved factors.

These study data were collected almost 5 years ago. However, SPARS is still highly relevant in its current context; a few modifications were introduced at the end of 2017 including two new indicators linked to malaria testing and treatment and to data quality for health information systems. No other supervision models have superseded SPARS. However, because of its well- documented influence, the MOH has now adapted a SPARS approach for laboratory, tuberculosis, and HIV/AIDS management. Although pharmacists were found to be very successful as MMS’s, it is not realistic to establish pharmacists at district level to implement SPARS in the near future in Uganda due to resource constraints. Steps have instead been taken to institute regional-level pharmacists to supervise MMS.

Despite these limitations, we believe that we have documented that SPARS is an effective strategy for improving MM at all levels of care within the government and PNFP sectors.

## Conclusions

Building capacity in MM at public and PNFP sector health facilities is critical to ensure high quality health services that rely on medicines availability and appropriate use. This study showed that the SPARS approach effectively improved medicines management practices in Uganda, with an improvement in overall performance of nearly 70% during the first year of supervision. We recognize that SPARS will evolve and that the performance assessment tool will change as health facility staff members become more adept in their skills. However, this study demonstrates the benefit of combining intervention strategies to change behaviors and performance in a low-resource health setting. We recommend monitoring SPARS scores for an extended time to assess further gains and to ascertain the program’s long-term cost-effectiveness.

## Additional files

Additional file 1: Multivariable models showing factors significantly associated with average changes in domain scores by level of care. (PDF 431 kb)

Additional file 2: Factors significant associated with attaining an adequate score. (DOCX 21 kb)

## Abbreviations

DHO: District Health Officer
EMHS: Essential medicines and health supplies
HC: Health center
HSD: Health sub-district
IQR: Interquartile range
MM: Medicines management
MMS: Medicines management supervisors
MOH: Ministry of Health
PNFP: Private not-for-profit
SPARS: Supervision, Performance Assessment and Recognition Strategy
