# Supplementary material for: Article 2: Longitudinal study assessing the one-year effects of supervision performance assessment and recognition strategy (SPARS) to improve medicines management in Uganda health facilities
Source: J Pharm Policy Pract. 2018 Jul 5;11:15. doi: 10.1186/s40545-018-0142-1 (PMC6033200; doi:10.1186/s40545-018-0142-1)
Supplement: Supplementary file 1 — Multivariable models showing factors significantly associated with average changes in domain scores by level of care. (PDF 431 kb) [file 40545_2018_142_MOESM1_ESM.pdf]

Additional file 1: Multivariable models showing factors significantly associated with average changes in domain scores by level of care

Dispensing quality	HC2		HC3		HC4/Hospital		All Facilities	
	Obs	Adj. Diff. (95%CI)	Obs	Adj. Diff. (95%CI)	Obs	Adj. Diff. (95%CI)	Obs	Adj. Diff. (95%CI)
**Region**								
Central	290	-					554	-
Western	493	-0.0 (-0.08 , 0.06)					886	**-0.0 (-0.08 , 0.03)**
Eastern	625	**0.1 (0.01 , 0.14)**					1088	**0.1 (0.02 , 0.12)**
Northern	231	**0.1 (0.06 , 0.22)**					383	**0.1 (0.05 , 0.18)**
**Ownership**								
Government	1434	-						
PNFP	205	-0.1 (-0.14 , 0.00)						
**Number of facilities designated MMS is tagged to**								
1-10					243	-		
11-15					47	0.0 (-0.20 , 0.12)		
16+					2	**0.7 (0.58 , 0.88)**		
**Highest level of education**								
Secondary/Diploma			859	-				
Bachelors/Master's degree			121	**-0.2 (-0.33 , -0.10)**				
**Baseline total Score**	1639	**-0.3 (-0.29 , -0.23)**	980	**-0.3 (-0.32 , -0.24)**	292	**-0.2 (-0.29 , -0.17)**	2911	**-0.3 (-0.29 , -0.24)**

Prescribing quality	HC2		HC3		HC4/Hospital		All Facilities	
	Obs	Adj. Diff. (95%CI)	Obs	Adj. Diff. (95%CI)	Obs	Adj. Diff. (95%CI)	Obs	Adj. Diff. (95%CI)
**Region**								
Central							554	-
Western							886	**0.2 (0.15 , 0.31)**
Eastern							1088	0.1 (-0.02 , 0.13)
Northern							383	**0.2 (0.11 , 0.29)**
**Number of facilities designated MMS is tagged to**								
1-10	578	-						
11-15	724	0.1 (-0.02 , 0.14)						
16+	337	**0.1 (0.00 , 0.21)**						
**Profession of responsible MMS**								
Pharmacist/dispensers			99	-	68	-	302	-
Clinician			545	**0.2 (0.09 , 0.38)**	148	-0.0 (-0.23 , 0.14)	1621	0.0 (-0.06 , 0.12)
Nurse/midwife			260	**0.3 (0.10 , 0.40)**	51	0.0 (-0.23 , 0.25)	789	0.0 (-0.07 , 0.12)
Supply/storekeeper			76	0.1 (-0.11 , 0.26)	25	**-0.3 (-0.52 , -0.05)**	199	**-0.2 (-0.28 , -0.04)**
**Have you received any feedback about your MMS activity reports from the DHO**								
No	240	-					417	-
Yes	1399	**0.2 (0.07 , 0.34)**					2494	**0.3 (0.16 , 0.36)**
**Baseline total Score**	1639	**-0.3 (-0.34 , -0.25)**	980	**-0.3 (-0.35 , -0.19)**	292	**-0.3 (-0.44 , -0.10)**	2911	**-0.3 (-0.36 , -0.28)**

**Stock management**	**HC2**		**HC3**		**HC4/Hospital**		**All Facilities**	
	**Obs**	**Adj. Diff. (95%CI)**	**Obs**	**Adj. Diff. (95%CI)**	**Obs**	**Adj. Diff. (95%CI)**	**Obs**	**Adj. Diff. (95%CI)**
**Region**								
Central	290	-						
Western	490	**-0.1 (-0.20 , -0.01)**						
Eastern	624	-0.1 (-0.15 , 0.04)						
Northern	231	-0.0 (-0.10 , 0.10)						
**Number of MMS supervising a facility**								
One MMS			821	-	196	-	2541	-
More than one MMS			159	**0.2 (0.02 , 0.34)**	96	**0.3 (0.04 , 0.50)**	366	**0.2 (0.07 , 0.27)**
**Number of health workers supervised at previous visit**								
One	571	-					686	-
More than one health worker	1064	**0.1 (0.03 , 0.22)**					2221	**0.1 (0.01 , 0.16)**
**Number of facilities designated MMS is tagged to**								
1-10	578	-						
11-15	723	**0.1 (0.02 , 0.15)**						
16+	334	0.1 (-0.03 , 0.15)						
**Profession of responsible MMS**								
Pharmacist/dispensers	135	-					302	-
Clinician	925	-0.1 (-0.19 , 0.03)					1618	-0.1 (-0.12 , 0.02)
Nurse/midwife	478	-0.1 (-0.17 , 0.05)					789	-0.0 (-0.10 , 0.06)
Supply/storekeeper	97	-0.2 (-0.32 , 0.01)					198	**-0.1 (-0.23 , -0.02)**
**Baseline total Score**	1635	**-0.4 (-0.42 , -0.31)**	980	**-0.4 (-0.45 , -0.34)**	292	**-0.3 (-0.42 , -0.20)**	2907	**-0.4 (-0.40 , -0.33)**

**Storage management**	**HC2**		**HC3**		**HC4/Hospital**		**All Facilities**	
	**Obs**	**Adj. Diff. (95%CI)**	**Obs**	**Adj. Diff. (95%CI)**	**Obs**	**Adj. Diff. (95%CI)**	**Obs**	**Adj. Diff. (95%CI)**
**Region**								
Central	290	-	208	-			554	-
Western	493	**0.1 (0.01 , 0.14)**	285	0.1 (-0.00 , 0.18)			886	**0.1 (0.01 , 0.13)**
Eastern	625	-0.1 (-0.14 , 0.02)	365	0.0 (-0.05 , 0.13)			1088	-0.0 (-0.08 , 0.03)
Northern	231	**0.2 (0.08 , 0.23)**	122	**0.2 (0.05 , 0.29)**			383	**0.2 (0.10 , 0.22)**
**Baseline total Score**	1639	**-0.3 (-0.34 , -0.27)**	980	**-0.3 (-0.36 , -0.28)**	292	**-0.3 (-0.33 , -0.18)**	2911	**-0.3 (-0.33 , -0.28)**

**Order reporting**	**HC2**		**HC3**		**HC4/Hospital**		**All Facilities**	
	**Obs**	**Adj. Diff. (95%CI)**	**Obs**	**Adj. Diff. (95%CI)**	**Obs**	**Adj. Diff. (95%CI)**	**Obs**	**Adj. Diff. (95%CI)**
**Region**								
Central	290	-	208	-			554	-
Western	493	**0.1 (0.02 , 0.24)**	285	**0.3 (0.21 , 0.47)**			886	**0.2 (0.11 , 0.27)**
Eastern	625	0.1 (-0.02 , 0.19)	365	**0.2 (0.07 , 0.31)**			1088	**0.1 (0.01 , 0.16)**
Northern	231	**0.2 (0.06 , 0.31)**	122	**0.3 (0.14 , 0.47)**			383	**0.2 (0.15 , 0.33)**
**MMS gender**								
Male					242	-	2390	-

Female					50	**-0.3 (-0.47 , -0.06)**	521	**-0.1 (-0.21 , -0.04)**
**Number of facilities designated MMS is tagged to**								
1-10	578	-			243	-		
11-15	724	0.1 (-0.02 , 0.14)			47	**0.3 (0.10, 0.49)**		
16+	337	**0.2 (0.05 , 0.27)**			2	0.3 (-0.26 , 0.95)		
**Profession of responsible MMS**								
Pharmacist/dispensers	135	-					302	-
Clinician	928	**-0.2 (-0.35 , -0.08)**					1621	**-0.1 (-0.20 , -0.02)**
Nurse/midwife	478	**-0.2 (-0.39 , -0.10)**					789	-0.1 (-0.16 , 0.06)
Supply/storekeeper	98	**-0.3 (-0.50 , -0.11)**					199	**-0.1 (-0.27 , -0.01)**
**Baseline total Score**	1639	**-0.3 (-0.40 , -0.29)**	980	**-0.4 (-0.41 , -0.32)**	292	**-0.4 (-0.48 , -0.30)**	2911	**-0.4 (-0.40 , -0.33)**
