# Supplementary material for: Article 2: Longitudinal study assessing the one-year effects of supervision performance assessment and recognition strategy (SPARS) to improve medicines management in Uganda health facilities
Source: J Pharm Policy Pract. 2018 Jul 5;11:15. doi: 10.1186/s40545-018-0142-1 (PMC6033200; doi:10.1186/s40545-018-0142-1)
Supplement: Supplementary file 2 — Factors significant associated with attaining an adequate score. (DOCX 21 kb) [file 40545_2018_142_MOESM2_ESM.docx]

**Additional file 2: Factors significantly associated with attaining an adequate score**

|  | **HC2** | | | **HC3** | | | **HC4 /Hospital** | | | | **All Facilities** | | |  |
| --- | --- | --- | --- | --- | --- | --- | --- | --- | --- | --- | --- | --- | --- | --- |
|  | **n/N*** | **%** | **Adj. hazard ratio (95%CI)** | **n/N*** | **%** | **Adj. hazard ratio (95%CI)** | **n/N*** | **%** | **Adj. hazard ratio (95%CI)** | **n/N*** | | **%** | **Adj. hazard ratio (95%CI)** |  |
| **Total number of facilities** | **159/681** | **23** |  | **80/416** | **19** |  | **34/125** | **27** |  | **273/1222** | | **22** |  |  |
| **Last visit after one year** |  |  |  |  |  |  |  |  |  |  | |  |  |  |
| Visits 1-3 | 45/359 | 13 | 1.0 | 24/230 | 10 | 1.0 | 12/72 | 17 | 1.0 | 81/661 | | 12 | 1.0 |  |
| Visit 4 or more | 114/322 | 35 | **3.0 (2.08 - 4.43)** | 56/186 | 30 | **3.0 (1.80 - 5.03)** | 22/53 | 42 | **3.0 (1.17 - 7.50)** | 192/561 | | 34 | **3.0 (2.29 - 3.93)** |  |
| **Region** |  |  |  |  |  |  |  |  |  |  | |  |  |  |
| Central | 18/133 | 14 | 1.0 | 7/92 | 8 | 1.0 |  |  |  | 27/250 | | 11 | 1.0 |  |
| Western | 50/224 | 22 | **2.2 (1.14 - 4.24)** | 31/145 | 21 | **3.1 (1.12 - 8.81)** |  |  |  | 94/421 | | 22 | **2.5 (1.49 - 4.06)** |  |
| Eastern | 52/226 | 23 | **1.9 (1.01 - 3.70)** | 27/118 | 23 | **3.6 (1.39 - 9.48)** |  |  |  | 90/379 | | 24 | **2.2 (1.36 - 3.58)** |  |
| Northern | 39/98 | 40 | **3.4 (1.73 - 6.83)** | 15/61 | 25 | **4.6 (1.31 - 16.18)** |  |  |  | 62/172 | | 36 | **3.7 (2.28 - 6.16)** |  |
| **Ownership** |  |  |  |  |  |  |  |  |  |  | |  |  |  |
| Government |  |  |  |  |  |  | 22/94 | 23 | 1.0 |  | |  |  |  |
| Private not for profit (PNFP) |  |  |  |  |  |  | 12/31 | 39 | **2.4 (1.10 - 5.08)** |  | |  |  |  |
| **Designated MMS at initial visit*** |  |  |  |  |  |  |  |  |  |  | |  |  |  |
| No | 41/208 | 20 | 1.0 |  |  |  |  |  |  |  | |  |  |  |
| Yes | 118/473 | 25 | **1.5 (1.01 - 2.24)** |  |  |  |  |  |  |  | |  |  |  |
| **No. facilities supervised by MMS** |  |  |  |  |  |  |  |  |  |  | |  |  |  |
| 1-10 |  |  |  | 41/176 | 23 | 1.0 |  |  |  |  | |  |  |  |
| 11-15 |  |  |  | 19/164 | 12 | **0.6 (0.34 - 0.98)** |  |  |  |  | |  |  |  |
| 16+ |  |  |  | 20/76 | 26 | 1.3 (0.68 - 2.51) |  |  |  |  | |  |  |  |
| **MMS professional training** |  |  |  |  |  |  |  |  |  |  | |  |  |  |
| Pharmacist/ dispenser | 89/402 | 22 | 1.0 |  |  |  |  |  |  | 144/717 | | 20 | 1.0 |  |
| Clinical officer | 26/56 | 46 | **2.0 (1.20 - 3.32)** |  |  |  |  |  |  | 47/121 | | 39 | **1.7 (1.20 - 2.51)** |  |
| Nurse/midwife | 39/185 | 21 | 0.9 (0.59 - 1.30) |  |  |  |  |  |  | 71/307 | | 23 | 1.1 (0.81 - 1.43) |  |
| Supplies officer | 5/38 | 13 | 0.5 (0.20 - 1.47) |  |  |  |  |  |  | 11/77 | | 14 | 0.6 (0.32 - 1.13) |  |
| **Received DHO feedback** |  |  |  |  |  |  |  |  |  |  | |  |  |  |
| No |  |  |  | 26/105 | 25 | 1.0 |  |  |  | 14/146 | | 10 | 1.0 |  |
| Yes |  |  |  | 38/218 | 17 | **3.8 (1.40 - 10.25)** |  |  |  | 195/782 | | 25 | **2.3 (1.30 - 4.00)** |  |
| **Baseline score** |  |  |  |  |  |  |  |  |  |  | |  |  |  |
| <= mean score | 57/390 | 15 | 1.0 | 33/225 | 15 | 1.0 |  |  |  | 103/681 | | 15 | 1.0 |  |
| > mean score | 97/267 | 36 | **2.3 (1.65 - 3.32)** | 45/169 | 27 | **1.9 (1.10 - 3.16)** |  |  |  | 161/491 | | 33 | **2.1 (1.60 - 2.75)** |  |
| * n=Number of facilities attaining adequate score; N=total number of facilities | | | | | | | | | | | | | | |
